# Weak and strong confinements in prismatic and cylindrical nanostructures

**DOI:** 10.1186/1556-276X-7-371

**Published:** 2012-07-05

**Authors:** Yuri V Vorobiev, Bruno Mera, Vítor R Vieira, Paul P Horley, Jesús González-Hernández

**Affiliations:** 1 CINVESTAV-Querétaro, Libramiento Norponiente 2000, Fracc. Real de Juriquilla, Querétaro, QRO, 76230, Mexico; 2 Centro de Física das Interacções Fundamentais, Instituto Superior Técnico, Universidade Técnica de Lisboa, Avenida Rovisco Pais, Lisbon, 1049-001, Portugal; 3 CIMAV Chihuahua/Monterrey, 120 Avenida Miguel de Cervantes, Chihuahua, CHIH, 31109, Mexico

## Abstract

Cylindrical nanostructures, namely, nanowires and pores, with rectangular and circular cross section are examined using mirror boundary conditions to solve the Schrödinger equation, within the effective mass approximation. The boundary conditions are stated as magnitude equivalence of electron's Ψ function in an arbitrary point inside a three-dimensional quantum well and image point formed by mirror reflection in the walls defining the nanostructure. Thus, two types of boundary conditions - even and odd ones - can be applied, when Ψ functions in a point, and its image, are equated with the same and the opposite signs, correspondingly. In the former case, the Ψ function is non-zero at the boundary, which is the case of a weak confinement. In the latter case, the Ψ function vanishes at the boundary, corresponding to strong quantum confinement. The analytical expressions for energy spectra of electron confined within a nanostructure obtained in the paper show a reasonable agreement with the experimental data without using any fitting parameters.

## Background

Nanostructures (NS) of different kinds have been actively studied during the last two decades, both theoretically and experimentally. A special interest was focused on quasi-one-dimensional NS such as nanowires, nanorods, and elongated pores that not only modify the main material's parameters, but are also capable of introducing totally new characteristics such as optical and electrical anisotropy, birefringence, etc. In particular, the existence of nanoscale formations on the surface (or embedded into semiconductor) result in quantum confinement effects. As the motion of the carriers (or excitons) becomes restrained, their energy spectra change, moving the permitted energy levels towards higher energies as a consequence of confinement. In the experimental measurements, such modification would be noticed as a blueshift of energy-related characteristics, such as, for example, the edge of absorption. This paper is dedicated to the theoretical investigation of confined particle problem, aiming to explain the available experimental data basing on geometry of corresponding nanoparticles present in the particular material. Here, we focus on elongated NS that can be approximated as prisms or cylinders with different shapes of cross section.

The theoretical treatment of NS is based on the solution of the Schrödinger equation, usually within the effective mass approximation [1-4], although for small NS, such approach can be questioned because the symmetry describing a nanoparticle may not inherit its shape symmetry but would rather depend on atomistic symmetry [5]. In addition, at small scale, it becomes necessary to take into account atomic relaxation and piezoelectric phenomena [6] that may strongly influence the energy states of confined particles and split their energy levels. The detailed consideration of these phenomena can be accounted using the pseudopotential method [7] introduced by Zunger's group that, after a decade, became a standard energy level model for detailed description of quantum dots. However, in cases when dimensions of nano-objects are large enough to validate the effective mass approximation, it is possible to obtain analytical solution to the problem of a particle confined within a quantum dot.

An important element of the quantum mechanical description is the boundary conditions; the traditional *impenetrable wall* conditions (1) are not always realistic and, (2) in many cases (depending on the shape of NS), could not be written in simple analytical form, thus complicating the further analysis. To overcome these problems, we proposed to use a mirrorlike boundary condition [8-10] assuming that the electron confined in an NS is specularly reflected by its walls acting as mirrors. In addition to a significant simplification of problem solution, this method favors the effective mass approximation.

Within the same framework, one can study pores as ‘inverted’ nanostructures (i.e., a void surrounded by semiconductor material) considering the ‘reflection’ of the particle's wave function from the surfaces limiting a pore. Thus, one will obtain essentially the same solution of the Schrödinger equation (and the energy spectrum) for both the pore and NS of the same geometry and size. A previous attempt to treat walls of a quantum system as mirrors in *quantum billiard* problem [11] yielded quite a complicated analytical form of the boundary conditions that made the solution of Schrödinger equation considerably more difficult.

In our treatment of the NS boundary as a mirror, the boundary condition equalizes absolute values of the particle's Ψ function in an arbitrary point inside the NS and the corresponding image point with respect to a mirror-reflective wall. Thus, depending on the sign of the equated Ψ values, one will obtain even and odd mirror boundary conditions. For the case of odd mirror boundary conditions (OMBC), Ψ functions in real point and its images should have the opposite sign, which means that the incident and reflected de Broglie waves cancel each other at the boundary. This case is equivalent to the impenetrable walls with vanishing Ψ function at the boundary, representing a ‘strong’ confinement case. However, some experimental data (see, e.g., [4]) show the evidence that a particle may penetrate the barrier, later returning into the confined volume. Thus, the wave function will not vanish at the boundary, and the system should be considered as a ‘weak’ confinement case as long as the particle flux through the boundary is absent. This case corresponds to even mirror boundary conditions (EMBC), when Ψ function in real point and its images are the same. Below, we analyze solutions of the Schrödinger equation for several cylindrical structures, using mirror boundary conditions of both types and making comparison of the energy spectra obtained with experimental data found in the literature.

## Methods

We start with the simplest case that could be easily treated on the basis of traditional approach - a NS shaped as a rectangular prism with a square base (with the sides *a* = *b* oriented along the axes *x* and *y*; the side *c* > *a* is set along the *z* direction). Assuming, as it is usually done in the literature, the absence of a potential inside the NS and separating the variables, we look for the solution of the stationary Schrödinger equation ΔΨ + *k*^2^ Ψ = 0 (where *k*^2^ = 2 *mE*/*ħ*^2^ and *m* being the particle's effective mass) as the product of plain waves propagating in both directions along the coordinate axes:

(1) $$\Psi =\prod_{j}\Psi_{j}\left(x_{j}\right)=\prod_{j}\left(A_{j}\exp(ik_{j}x_{j}\right)+B_{j}\exp(-ik_{j}x_{j}))$$

For this case, the even mirror boundary conditions are as follows [10]:

(2) $$\Psi \left(x,y,z\right)=\Psi (-x,y,z)=\Psi (x,-y,z)=\Psi (x,y,-z)=\Psi (2a-x,y,z)=\Psi (x,2b-y,z)=\Psi (x,y,2c-z)$$

That renders the following solution (Equation 1) of the Schrödinger equation:

(3) $$\Psi \left(x,y,z\right)=A\;\text{cos}\;k_{\text{x}}x\;\text{cos}\;k_{\text{y}}y\;\text{cos}\;k_{\text{z}}z$$

with wave vector components

(4) $$k_{\text{x}}a=\pi n_{\text{x}}\text{,}\;k_{\text{y}}b=\pi n_{\text{y}}\;\text{and}\;k_{\text{z}}c=\pi n_{\text{z}}$$

It gives the following energy spectrum:

(5) $$E=\frac{h^{2}}{8m}\left(\frac{n_{x}^{2}}{a^{2}}+\frac{n_{y}^{2}}{b^{2}}+\frac{n_{z}^{2}}{c^{2}}\right)\text{or}\frac{h^{2}}{8m}\left(\frac{n_{x}^{2}+n_{y}^{2}}{a^{2}}+\frac{n_{z}^{2}}{c^{2}}\right)$$

The odd mirror boundary conditions are obtained from Equation 2 by inverting the sign of the left-hand-side function. The solution will then be as follows:

(6) $$\Psi \left(x,y,z\right)=B\;\text{sin}\;k_{\text{x}}x\;\text{sin}\;k_{\text{y}}y\;\text{sin}\;k_{\text{z}}z$$

The wave vector components will be the same as that presented in Equation 4, yielding the same energy spectrum (Equation 5). Using the traditional impenetrable wall boundaries, one will also obtain the solution in the form (Equation 6) that coincides with the OMBC solution that has a vanishing Ψ function at the boundary. Therefore, the energy spectrum is the same for both types of mirror boundary conditions and impenetrable wall boundary, although the solutions themselves are not equal. In [7], we demonstrated that for NS of spherical shape, the energy spectrum found with EMBC (weak confinement) is different from that corresponding to impenetrable walls conditions.

From Equation 5, it is evident that the energy spectrum of prismatic (cylindrical) NS is a sum of the spectra corresponding to the two-dimensional cross-section NS (a square with side length *a*) and the one-dimensional wire of length *c*. In a similar manner, the spectrum for cylinders with other cross-section shapes can be constructed using the solutions for two-dimensional triangular or hexagonal structures analyzed previously [8,9]. Below, we present the analysis of cylindrical NS.

Let us consider a nanostructure with a circular cross section of diameter *a* and cylinder height *c*. The solution of the problem using a traditional approach can be found in [12,13]. In our case, we make variable separation in cylindrical coordinates:

(7) $$\Psi \;\left(r,\varphi ,z\right)=AF\left(r\right)\text{exp}(ip\varphi )[B\text{exp}\left(ikz\right)+C\text{exp}(-ikz)],\text{ with integer}\;p=0\text{,}\pm 1\text{,}\pm 2$$

We note that the value of *p* defines the angular momentum: *L* = *pħ.* In the case of EMBC, one can apply mirror reflection from the base, which gives *B* = *C*, resulting in the following wave function:

(8) $$\Psi (r,\varphi ,z)=AF\left(r\right)\text{exp}\left(ip\varphi \right)\text{cos}kz$$

Strong confinement (OMBC) gives *B* = −*C*, which introduces sin*kz* instead of cos*kz* in Equation 7A.

The radial function *F*(*r*) is the solution of the following radial equation:

(9) $$\frac{d^{2}F(r)}{dr^{2}}+\frac{1}{r}\frac{dR}{dr}+\left(k^{2}-\frac{p^{2}}{r^{2}}\right)F(r)=0$$

It is Bessel's differential equation regarding the variables *kr*, the solution of which is given by the cylindrical Bessel function of integer order |*p*|: *J*_|*p*|_(*kr*); with, *k* = *ħ*^−1^(2*mE*_*n*_)^1/2^. Here, *m* is the effective mass of the particle, and *E*_*n*_ is the quantized kinetic energy corresponding to the motion in two-dimensional circular quantum well. The total energy consists of energy contribution for the motion within cross-section plane and along the vertical axis *z*: *E* = *E*_*n*_ + *E*_z_.

**Figure 1:**
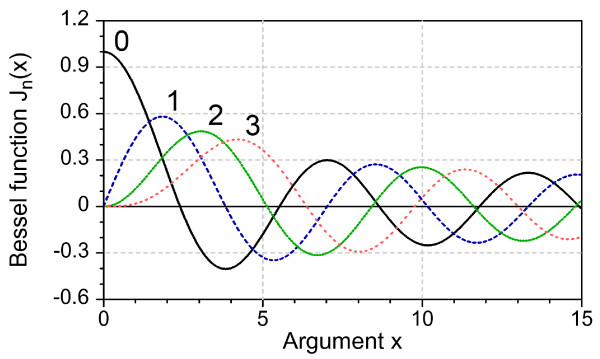
**Cylindrical Bessel functions*J***_***n***_**(*x*).** Curve numbers correspond to order *n.*

The energy *E*_n_ depends on the values of *k* and is obtained using boundary conditions. In the traditional case of impenetrable walls, the Ψ function vanishes at the boundary so that the energy values are determined by the roots (nodes) of the cylindrical Bessel function (see Figure 1 for different order numbers *n*, and also Table 1). The same situation will take place for OMBC, yielding zero wave function at the boundary so that the nodes *q*_|*p*|*i*_ of the Bessel function will define the energy values.

**Table 1:** Argument values at nodes and extremes of cylindrical Bessel function

**|*p*|**	***q***_**|*p*|1**_	***t***_**|*p*|1**_	***q***_**|*p*|2**_	***t***_**|*p*|2**_	***q***_**|*p*|3**_	***t***_**|*p*|3**_	***q***_**|*p*|4**_	***t***_**|*p*|4**_
0	2.4	0	5,5	3.713	8.5	7.10	11.6	10.15
1	0	1.625	3.7	5.375	6.9	8.55	10.25	11.6
2	0	2.92	5.11	6.775	8.4	10.0	11.65	13.15
3	0	4.325	6.4	8.1	9.85	11.4	13.2	14.2

If the EMBC are used, the situation becomes different since the function values in the points approaching the boundary of the nanostructure should match those in the image points, making the boundary to correspond to the extremes of the Bessel function (which was strictly proved for the spherical quantum dots (QDs) [10]).

Table 1 gives several values of the Bessel function argument *kr* corresponding to the function nodes (*q*_|*p*|*i*_) and extremes (*t*_|*p*|*i*_) calculated for function orders 0, 1, 2, and 3.

At the boundary, *r* = *a*/2; therefore, the corresponding value of *k* is 2*q*_|*p*|i_/*a* for OMBC and 2 *t*_|*p*|i_/*a* for EMBC. The energy spectrum for a particle confined in a circular-shaped quantum well is as follows:

(10) $$E_{n}=\frac{2\hbar^{2}}{ma^{2}}S_{|p|i}^{2}=\frac{\hbar^{2}}{2\pi^{2}ma^{2}}S_{|p|i}^{2}$$

Here, the parameter *s*_|*p*|i_ takes the values of *q*_|*p*|i_ for OMBC (strong confinement) and *t*_|*p*|i_ for EMBC (weak confinement).

The quantization along the *z* axis for both the boundary condition types will be $E_{z}=\frac{h^{2}}{8m}\frac{n_{z}^{2}}{c^{2}}$, yielding the total energy

(11) $$E=\frac{h^{2}}{2m}\left(\frac{S_{|p|i}^{2}}{\pi^{2}a^{2}}+\frac{n^{2}}{4c^{2}}\right)$$

In the case of EMBC, the ground state (GS) energy will be obtained with *t*_11_ = 1.625:

(12) $$E_{\text{GS}}=\left(h^{2}/2m\right)\left(0.268/a^{2}+\;1/4c^{2}\right)$$

In the OMBC case, the GS will be determined by the smallest *q* value of 2.4:

(13) $$E_{\text{GS}}=\left(h^{2}/2m\right)\left(0.584/a^{2}+\;1/4c^{2}\right)$$

Equations 10, 11, and 11A can be used for the analysis of optical processes in the NS discussed. In particular, blueshift in exciton ground state can be found from Equations 11 and 11A if one substitutes a reduced exciton mass in place of particle mass *m*. Using Equation 10, it is possible to obtain in a similar way the energies corresponding to the higher excited states.

**Figure 2:**
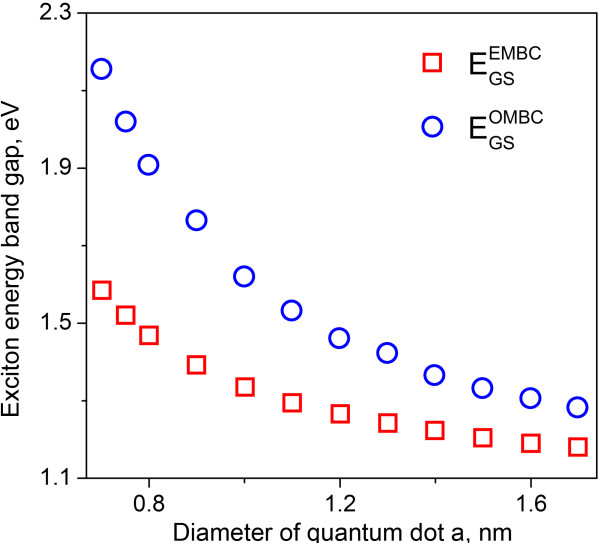
**Dependence of ground state energy on diameter of a cylindrical nanostructure.** The plot shows the data obtained with odd and even mirror boundary conditions for an NS with parameters of silicon.

For long NS with sufficiently large *c*, the second term in energy does not affect the GS. Thus, the solution for cylindrical NS based on even mirror boundary conditions EMBC (weak confinement) gives the GS shift due to quantum confinement that is (2.4/1.625)^2^ = 2.18 times smaller than the value obtained for the strong confinement case. In the case of spherical QD [10], the difference was four times. It is reasonable that for strong confinement, the blue shift value exceeds that obtained for the weak confinement case. To illustrate this, we present in Figure 2 the comparison of ground state energy obtained with OMBC and EMBC (using Equations 11 and 11A) on NS diameter for a cylindrical quantum well with parameters of silicon (effective mass for electron 0.26 and 0.49 for a hole, which corresponds to reduced exciton mass of 0.17; bandgap is 1.1 eV for 300 K). As one can see from the figure, the difference of the exciton bandgap scales down with increase of the NS diameter, with invariably higher values observable for the strong confinement case described by OMBC.

The choice of OMBC or EMBC has to be made taking into account the probability of electron tunneling through the walls forming the nanostructure. One can expect that in the case of isolated NS strong confinement (OMBC), approximation will be more appropriate, whereas for NS surrounded by other solid or liquid media (core-shell QDs [10] and pores in semiconductor media), weak confinement with EMBC should be used.

## Results and discussion

Considerable scientific interest has been attracted to semiconductor nanorods (nanowires) and cylindrical pores. Let us mention here publications dealing with arrays of cylindrical pores in sapphire [14], ZnO nanorods grown within these pores [15], as well as CuS and In_2_O_3_ nanowires. Usually, the experiments report on relatively large structures measuring 30 nm or more in diameter. As one can see from Equations 11 and 11A, in these cases, the expected blueshift will be about 0.01 eV or less for both the weak and strong confinements. Nevertheless, there exists literature data referring to nanorods of sufficiently small diameter for a pronounced confinement effect.

A paper [16] reports on CdS nanorods with a diameter of 5 nm and a length of 40 nm embedded into a liquid crystal. The authors study the optical anisotropy caused by the alignment of the nanorods. To determine it, they measure polarization of photoluminescence due to electron–hole recombination, reporting that the spectral maximum of luminescence is located at 485 nm (2.56 eV), which exceeds the bandgap of the bulk CdS by 0.14 eV. Taking the electron effective mass in CdS [17] as 0.16 *m*_0_ and hole effective mass 0.53 *m*_0_, one can find the reduced mass *μ* = 0.134 *m*_0_ and the blueshift 0.12 eV using Equation 11, which agrees reasonably with the experiment. As CdS nanostructure is surrounded by liquid crystal media, we were using the EMBC or weak confinement approximation.

Another study [18] is focused on the optical properties of CuS nanorods measuring 6 to 8 nm in diameter and 40 to 60 nm in length; the authors report definite blueshift of fundamental absorption edge. Alas, we found no data on the effective masses for CuS, so it was not possible to make numerical comparison with the theory.

**Figure 3:**
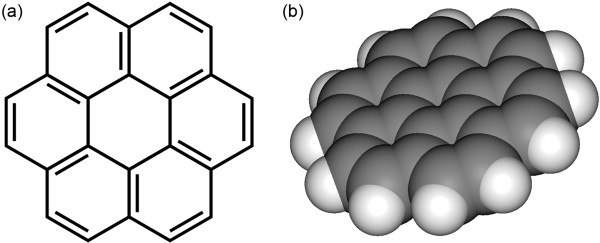
Coronene molecule (a) formula and (b) computer-rendered three-dimensional image.

A particular example of cylindrical QDs is presented by quasi-circular organic molecules like coronene C_24_H_12_ (see Figure 3). In this case *c* < <*a*, which makes the second term in Equations 10, 11, and 11A very large even for *n*_z_ = 1, meaning that it has no contribution to the optical properties of the molecule in visible light because the transitions between the states with different *n*_z_ will correspond to radiation in deep ultraviolet. Therefore, the spectrum is defined by the first term in Equations 10 and 11 that essentially replicates the solution obtained for the case of a long cylinder.

Another paper [19] presents the experimental data concerning the optical properties of coronene molecules in tetrahydrofuran (THF) solution. Since the molecules are submerged into media, we expect that weak confinement/EMBC will be most appropriate for solution of the problem. Strong absorption lines were registered at photon energies of 4.1 to 4.3 eV, with weaker absorption down to 3.5 eV. To use our methodology, one should first determine the diameter *a* of a circle embracing the molecule with its 12 atoms of carbon (Figure 3).

The C-C bond length in coronene is *d* = 1.4 AǺ, which corresponds to the side of a hexagon. Thus, one would have *a* = $d\sqrt{28}$ = 0.741 nm. Taking in (Equation 11) *m* as free electron mass and using only the first term, we obtain the ground state energy *E*_GS_ *=* 0.73 eV. The higher energy states (Equation 10) will be defined by the values of *s*_|*p*|i_ = *t*_|*p*|i_ equal to 2.92, 3.713, 4.30 etc. The corresponding energies are 2.353, 3.805, and 5.1 eV that result in transition energies 1.62, 3.1, and 4.37 eV. The first value is out of the spectral range investigated in [19]; the other two could reasonably fit the absorption observed.

If we attempt to treat the case on the basis of strong confinement approximation (OMBC), one should use the *q*_|*p*|i_ values in the formulas (Equations 10 and 11A), yielding the ground state of 1.591 eV and excited states at 3.78, 7.21, and 8.35 eV. Therefore, the transition energies would be 2.19, 5.62, and 6.76 eV which have nothing in common with the experimental values, proving that the previous conclusion to use EMBC based on the fact that coronene molecules are embedded into THF medium was the right one.

Yet, another paper [20] is devoted to studying coronene-like nitride molecules with the composition N_12_*X*_12_H_12_, where *X* can be B, Al, Ga or In. Depending on *X*, the bond length will vary, giving different values of well diameter *a*. The authors of [20] give the transition energies between the ground state and the first excited state, corresponding to HOMO-LUMO transition *E*_HL_. For these isolated molecules, the strong confinement case/OMBC is expected to be appropriate. The bond lengths and *E*_HL_ values reported in [20] are listed in Table 2 together with values of *a* calculated from bond length and the transition energies Δ*E* found using the expression (Equation 10) with corresponding *q* values. One can see that Δ*E* values are reasonably close to the experimental *E*_HL_. Solution of the same problem using weak confinement/EMBC results in large discrepancies that fails to explain the experimental data, confirming the correctness of the decision to choose OMBC for isolated molecules.

**Table 2:** The lowest transition energies in coronene-like molecules

**Material**	***d*(Å)**	***a*(nm)**	**Δ*E*(eV)**	***E***_**HL**_**(eV) **[17]**]**
BN	1.44	0.762	6.351	5.18
AlN	1.79	0.95	4.11	4.59
GaN	1.84	0.974	3.88	3.94
InN	2.06	1.09	3.1	2.33

## Conclusions

Theoretical description of prismatic and cylindrical nanostructures (including pores in semiconductor) is made using two types of mirror boundary conditions for solution of the Schrödinger equation, resulting in simple analytical procedure to obtain wave functions that offer reasonably good description of optical properties of nanostructures of various shapes. The expressions for energy spectra are defined by the geometry and dimensions of the nanostructures. The even mirror boundary conditions correspond to weak confinement that is applicable for the cases when the nanostructure is embedded into another media (which is especially true for a case of a pore) that enables tunneling through the boundary of the nanostructure. In contrast, odd mirror boundary conditions are more appropriate in the treatment of isolated nanostructures where strong confinement exists. Both cases are illustrated with experimental data, proving good applicability of the corresponding type of boundary conditions.

## Competing interests

The authors declare that they have no competing interests.

## Authors’ contributions

YVV and VRV performed calculations and drafted the manuscript. BM helped in drafting the manuscript. PPH and JG-H provided helpful discussions and improvement for the manuscript. All authors read and approved the final manuscript.
